# Comparison and calibration of transcriptome data from RNA-Seq and tiling arrays

**DOI:** 10.1186/1471-2164-11-383

**Published:** 2010-06-17

**Authors:** Ashish Agarwal, David Koppstein, Joel Rozowsky, Andrea Sboner, Lukas Habegger, LaDeana W Hillier, Rajkumar Sasidharan, Valerie Reinke, Robert H Waterston, Mark Gerstein

**Affiliations:** 1 Department of Molecular Biophysics and Biochemistry, Yale University, New Haven, CT 06520, USA; 2 Program in Computational Biology and Bioinformatics, Yale University, New Haven, CT 06520, USA; 3 Department of Genome Sciences, University of Washington School of Medicine, Seattle, Washington 98195, USA; 4 Department of Genetics, Yale University School of Medicine, New Haven, Connecticut 06520, USA

## Abstract

**Background:**

Tiling arrays have been the tool of choice for probing an organism's transcriptome without prior assumptions about the transcribed regions, but RNA-Seq is becoming a viable alternative as the costs of sequencing continue to decrease. Understanding the relative merits of these technologies will help researchers select the appropriate technology for their needs.

**Results:**

Here, we compare these two platforms using a matched sample of poly(A)-enriched RNA isolated from the second larval stage of *C. elegans*. We find that the raw signals from these two technologies are reasonably well correlated but that RNA-Seq outperforms tiling arrays in several respects, notably in exon boundary detection and dynamic range of expression. By exploring the accuracy of sequencing as a function of depth of coverage, we found that about 4 million reads are required to match the sensitivity of two tiling array replicates. The effects of cross-hybridization were analyzed using a "nearest neighbor" classifier applied to array probes; we describe a method for determining potential "black list" regions whose signals are unreliable. Finally, we propose a strategy for using RNA-Seq data as a gold standard set to calibrate tiling array data. All tiling array and RNA-Seq data sets have been submitted to the modENCODE Data Coordinating Center.

**Conclusions:**

Tiling arrays effectively detect transcript expression levels at a low cost for many species while RNA-Seq provides greater accuracy in several regards. Researchers will need to carefully select the technology appropriate to the biological investigations they are undertaking. It will also be important to reconsider a comparison such as ours as sequencing technologies continue to evolve.

## Background

Unbiased, high-throughput analytical methods are essential tools for identifying novel RNAs, discerning alternative splicing isoforms, and determining gene expression levels. Tiling arrays have been the investigative tool of choice and continue to lead to novel discoveries. They effectively identify novel transcribed regions [1-4] and quantify expression levels [5]. They were recently employed in the discovery of ubiquitous bidirectional promoters in yeast [6], and microarrays tiling certain regions of the human genome were used to find new sets of conserved lincRNAs [7].

On the other hand, it is becoming increasingly apparent that massively parallel transcriptome sequencing has distinct advantages over arrays. RNA-Seq inherently provides single nucleotide resolution and in some contexts requires only minimal *apriori *knowledge of the genome, while tiling arrays exhibit cross-hybridization and have a limited dynamic range of detection [8,9]. There has been a recent explosion in the use of RNA-Seq to globally survey transcriptomes, including *S. cerevisiae *[9], *S. pombe *[10], *B. anthracis *[11], *B. cenocepacia *[12], *C. elegans *[13], *A. thaliana *[14], *M. musculus, H. sapiens *[8], and others. It has excelled at determining exon boundaries and as a corollary, at detecting and quantifying alternative splicing [9,13,15-17]. Previous studies have used RNA-Seq exclusively [9] or in conjunction with tiling arrays [10] to map the 5' and 3' exon boundaries of *S. cerevisiae *and *S. pombe*, respectively. Strikingly, 86% of the 5' UTR boundaries of *S. cerevisiae *genes have been identified without use of a prior annotation [18]. It has even been effective at the single cell level and detects significantly more transcripts than single cell tiling array methods [19].

However, tiling arrays remain more cost effective for many species despite a dramatic reduction in the cost of sequencing in recent years. Our consequent belief that both tiling arrays and RNA-Seq will continue to be used in transcriptomics motivated us to objectively compare their performance, to understand how cross-platform results can be interpreted, and to develop a method for calibrating tiling array analysis based on RNA-Seq data. Previous studies have compared DNA microarrays with massively parallel signature sequencing (MPSS) [20], tiling arrays with MPSS [21], and gene expression arrays with Solexa/Illumina sequencing [22,23], but our work is the first to compare tiling arrays with deep sequencing technology on a matched sample. This is especially relevant because tiling arrays, unlike expression arrays, can detect novel transcripts and so are a more realistic alternative to sequencing.

In this work, we quantitatively assess tiling array and RNA-Seq performance using a matched sample of poly(A)-enriched *C. elegans *RNA from the L2 larval stage. We also used two total RNA samples from the L2 and young adult stages for differential expression analysis. Our comparisons are of two types: correspondence between the two platforms, and their relative performance compared to a gold standard set. We find the raw signals to be generally well correlated, and the transcriptionally active regions (TARs) predicted by the two platforms are broadly similar. However, RNA-Seq's greater dynamic range of expression allows more differentially expressed genes to be identified. Furthermore, comparison to known exons shows that RNA-Seq predicts exon boundaries more accurately, and a receiver operating characteristic (ROC) analysis against a gold standard set shows that RNA-Seq provides better sensitivity at lower false positive rates (FPR). These results are qualitatively as expected and we are able to quantitate the differences.

Since reads are costly, we also investigated the depth of sequencing required for the two platforms' performance to be comparable. We found that 4 million reads are required for RNA-Seq to achieve the same sensitivity, at a given FPR, as 2 replicate tiling arrays. This corresponds to a previous finding that 4 million reads are required to detect 80% of expressed genes in *S. cerevisiae *[9]. However, the experimental goals can affect this number significantly. In other work on murine embryonic stem cells, eighty million reads were required before the detection of unique start sites plateaus [24].

Next, we investigated cross-hybridization effects in tiling arrays by comparing expression levels for transcriptional regions with those from paralogous pseudogenes and "nearest neighbor" regions. If a region's expression level is affected by cross-hybridization we expect these values to be correlated, and indeed find this to be the case for many annotated regions. The same analysis with RNA-Seq data does not show such a correlation, although mapping ambiguities are an analogous problem in RNA-Seq data [13]. Finally, we considered the problem of calibrating tiling array analysis using RNA-Seq as a gold standard set. We describe a method to optimize the parameters of the maxgap/minrun segmentation algorithm and then assign an adjusted confidence score to each TAR by using the RNA-Seq data.

## Results

### Data sets

The tiling array analysis was carried out using the Affymetrix *C. elegans *Tiling 1.0R Array containing 25-mer perfect match (PM) and mismatch (MM) probes tiled over the *C. elegans *genome. The vast majority of adjacent probes either slightly overlap or have a gap between them of a few base pairs. RNA-Seq was carried out using an Illumina cluster station and 1G analyzer, and we aligned reads to the WS170 build of the *C. elegans *genome and splice junction databases using MAQ [25] and cross_match (P. Green, http://www.phrap.org/phredphrap/phrap.html; [26]), respectively. Extensive details about the RNA-Seq data and methods are provided by Hillier et al. [13].

RNA samples were prepared and shipped frozen on dry ice between the two labs conducting the array and sequencing work. The main sample studied was poly(A)-enriched RNA from the L2 larval stage of *C. elegans*; we notate this L2-poly(A). We sequenced poly(A)-enriched RNA because the reads would otherwise be overwhelmed by rRNA and tRNA, which together comprise >95% of total RNA [27]. Recent methods overcome this requirement by depleting ribosomal RNA [28]. Although arrays work well with total RNA, we hybridized the same poly(A)-selected RNA to permit direct comparison between the platforms. Two biological replicates were hybridized on the tiling array and about 32 million aligned reads (from a yield of 116 M from 12 lanes) were obtained by RNA-Seq.

In addition, for a differential expression analysis of genes, we also prepared a young adult (YA) sample, which we compared to the L2 stage. In this case, total RNA was used for both stages in the array (notated L2-tot and YA-tot) and poly(A)-enriched RNA was used for both sequenced samples. For these samples, the array data is comprised of 3 replicates, and RNA-Seq generated about 28 million aligned reads. Our ROC analyses required a set of annotated transcribed (positive) and non-transcribed (negative) regions, for which we utilized a high confidence subset of the WormBase annotation as extracted by Hillier et al. [13]. This annotation covers only 45% of base pairs because it does not simply consider unannotated regions as negatives. Rather, each base pair is marked as either exonic, intronic, or intergenic only when this can be claimed with high confidence (Additional file 1). We refer to this as our "gold standard" annotation set.

This does not however demarcate genes and exons. For those analyses requiring a set of exons grouped into genes, we began with the WormBase annotation and then created "composite gene models" to avoid double counting isoforms. This was done by taking the union of exonic base pairs for each group of transcripts arising from the same gene. For example, if one isoform has exons 1, 2, and 3, and another has exons 3 and 4, the composite gene will contain exons 1, 2, 3, and 4. Also, overlapping exons get merged and we term the resulting contiguous regions "composite exons" (Additional file 1). Importantly, these annotations serve as an independent verification of our data since neither tiling array nor sequencing based evidence is included in them.

Many of our analyses required us to segment the RNA-Seq and tiling array signals into TARs. We employed the maxgap/minrun algorithm [29,30], and, as discussed in a later section, chose parameters affecting this algorithm by optimizing against the gold standard set.

### Correlating RNA-Seq and Tiling Array Signals

The first analysis we undertook was a direct comparison of the signal from the two platforms. A tiling array's signal is defined as an intensity value for each probe. The PM minus MM values are computed for all replicates, and the replicates' signals are combined using pseudomedian smoothing over a window of 110 bp [30]. The signal of RNA-Seq data is defined as a count of the number of reads overlapping at each base pair. Neither replicates nor smoothing were deemed necessary since it has high signal-to-noise ratio. We conclude this by observing very high correlations (≥ 0.98) between "pseudo-replicates" that we constructed by downsampling, selecting random subsets of all reads available (Additional file 1: Table S1).

**Figure 1:**
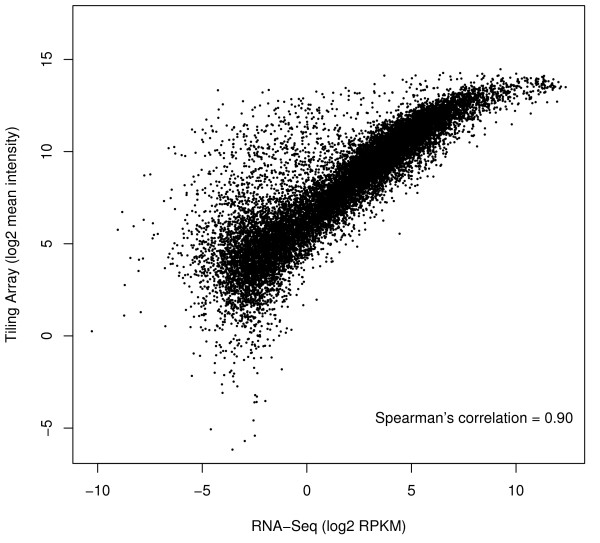
**Correlation of RNA expression levels between RNA-Seq and tiling array platforms**. Each point represents a gene from the composite model. RNA-Seq expression levels per gene were measured using RPKM, and tiling array levels were measured using the mean intensity of probes falling within composite exons. The Spearman's coefficient is 0.90, indicating that the platforms correlate well on identical samples. The disproportionate number of genes in the upper left likely represents cross-hybridization.

From the L2-poly(A) signal for both platforms, we computed an expression level for annotated genes by taking the mean of exonic probe values in the case of the array and the reads per kilobase million (RPKM) in the case of RNA-Seq. RPKM is a better quantitation of RNA-Seq expression because it accounts for molar concentration and transcript length [8] (Methods). Figure 1 shows that expression levels correlate well for the two platforms (Spearman's correlation = 0.90), significantly higher than the Pearson correlations ranging from 0.40 - 0.52 reported previously between MPSS and expression arrays [20]. The logarithmic nature of the curve likely arises due to saturation of the microarray's scanner signal [31]. Furthermore, in the top left, we note an abundance of genes with high average microarray intensities but low read coverage by sequencing. This is likely due to cross-hybridization and is discussed in a later section.

### Differential Expression

Next, we examined the ability of both technologies to identify differentially expressed genes between the L2 and young adult (YA) life stages. The Wilcoxon rank sum test was utilized followed by multiple hypothesis correction [32-34], and we required the corrected q-value to be less than 0.01 for a gene to be called differentially expressed.

The Wilcoxon test requires the two samples being compared to have an equal number of data points, which is not the case between array and sequencing signals; there are fewer probes in a gene than base pairs. We resolve this by converting the RNA-Seq signal to values on a "pseudoarray". A pseudoarray provides intensity levels for each tiling array probe, except the intensity is computed from reads falling within the probe's coordinates. In this way, the RNA-Seq data mirrors the tiling array. We found that this has only a minute effect on signal quality for analyses not dependent on base pair resolution (Additional file 1: Figure S1).

**Figure 2:**
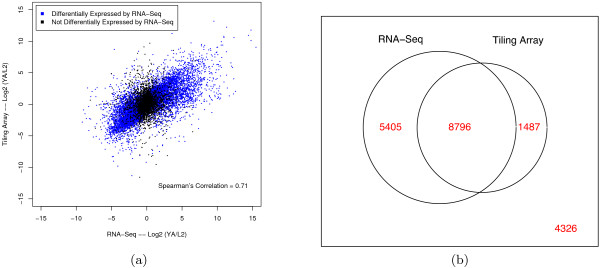
**Differential expression of genes between the L2 and YA stages**. (a) Correlation of log2(YA/L2) ratios between RNA-Seq and tiling arrays. Differential expression was determined using the nonparametric Wilcoxon rank sum test. Black: not significantly differentially expressed between samples. Blue: significantly differentially expressed (*q *≤ 0.01). The ratio of expression levels is well-correlated, but RNA-Seq has a larger dynamic range. (b) Venn diagram of genes called differentially expressed by each platform. There is significant overlap (8,976) between the two platforms, but more genes were called differentially expressed by RNA-Seq (14,201) than by tiling arrays (10,283), likely reflecting its greater dynamic range. A total of 4,326 genes were not called differentially expressed by either technology.

Figure 2a plots the log2 ratio of expression between YA and L2 for both platforms. Although the ratio was reasonably correlated (Spearman's coefficient = 0.71), we note that the dynamic range of differential expression as measured by tiling arrays was much less than that of RNA-Seq. Specifically, RNA-Seq is able to detect larger fold differences, probably owing to the scanner signal's saturation for arrays.

We found 14,201 differentially expressed genes by RNA-Seq, and 10,283 by the tiling array data. The Venn diagram in Figure 2b shows that 86% of those called differentially expressed by the array were also detected by RNA-Seq. However, 38% of those called by RNA-Seq were not detected as differentially expressed by the array.

**Figure 3:**
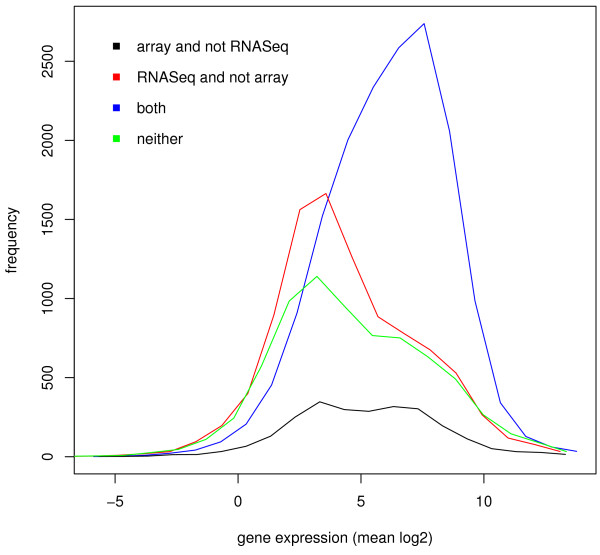
**Histograms of gene expression levels**. Four disjoint sets of genes are considered, those differentially expressed: by arrays but not RNA-Seq (black), by RNA-Seq but not arrays (red), by both platforms (blue), and by neither platform (green). Genes detected by just one platform (black, red) have lower expression than those detected by both (blue).

Four regions in the Venn diagram describe those genes differentially expressed: by RNA-Seq but not arrays, by arrays but not RNA-Seq, by both platforms, and by neither platform. Figure 3 depicts histograms of gene expression levels based on array data for each of these categories. We collected the values for young adult and L2 samples into one pool. It is apparent that genes found to be differentially expressed by only one platform have lower expression than those detected by both. Both RNA-Seq and tiling arrays selectively detect differential expression in genes expressed at lower levels, and as expected low-expression genes are often not detected as differentially expressed by either platform. The results are similar if the analysis is based on expression levels computed from the RNA-Seq data (Additional file 1: Figure S5).

### GC Content Bias

From the array signal, we found that the expression level of a gene is significantly correlated with its GC content (Spearman's coefficient = 0.30, Kolmogorov-Smirnov test: *p *< 10^-15^, *D *= 0.1522; Additional file 1: Figure S2a). This bias is not wholly unexpected. Microarrays depend on hybridization, and guanosine-cytosine pairs have a free energy of binding that is roughly 2 kcal/mol stronger than that of adenine-thymine [35]. Thus, probes that tile a gene with higher GC content will likely bind to its complementary cDNA more tightly, potentially skewing the results.

Interestingly, we also found a smaller, but still significant, GC bias in the RNA-Seq data as well (Spearman's coefficient = 0.16, Kolmogorov-Smirnov test: *p *< 10^-15^, *D *= 0.0991; Additional file 1: Figure S2b). This could reflect some bias in the amplification procedure, an intrinsic bias in expressed genes having higher GC content, or some combination of the two.

### Exon boundary detection

The Affymetrix tiling array used in this study has probes that are 25 bp in length. As a result, we cannot expect feature boundaries to be detected with an accuracy much higher than this. RNA-Seq data however potentially detects features with single base pair resolution. We investigated the relative ability of the two platforms to detect feature boundaries by quantifying the overlap between every exon in the gold standard set and the corresponding TARs. The offset is defined as positive or negative if the TAR boundary extends beyond or falls short, respectively, of the exon boundary. We excluded TARs that overlap with more than one annotated exon.

**Figure 4:**
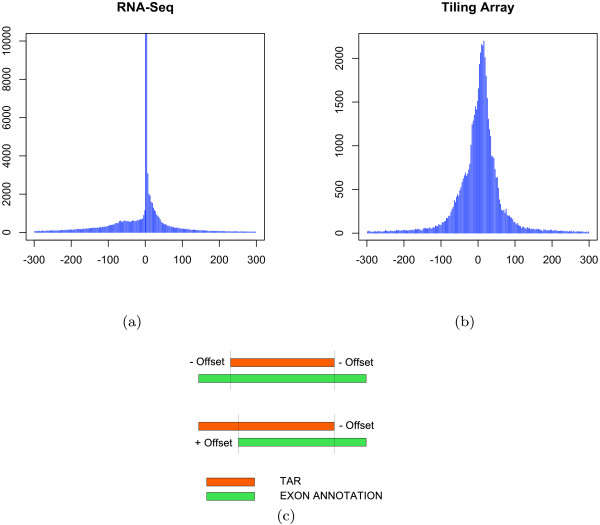
**Exon boundary detection for tiling array and RNA-Seq**. For every TAR, we computed its offset from its overlapping exon (excluding those that did not overlap with exactly one exon). (a) RNA-Seq has a median offset of 0 bp and a median absolute deviation of 2 bp, whereas (b) the tiling array has a median offset of 7 bp and a median absolute deviation of 25 bp. (c) Pictorial representation of how offsets were calculated. A negative offset means the TAR (orange) falls short of the exon (green) boundary and a positive offset means the TAR extends beyond the exon.

Figure 4 shows the resulting distribution of offsets for both technologies. It is evident that RNA-Seq provides much higher accuracy, with a median offset of 0 base pairs, whereas the tiling array exons have a median offset of 7 base pairs. Interestingly, the median absolute deviation of RNA-Seq is 2 base pairs, and the corresponding deviation of tiling arrays is 25 base pairs, corresponding closely to the expected resolution from each platform.

We also investigated the possibility of a 3' mapping bias in RNA-Seq [8] by plotting the number of TARs that overlap at each point along exons from their 5' to 3' end (Additional file 1: Figure S3).

Unsurprisingly, we did not find any bias because random hexamers were used to prime cDNA synthesis in conjunction with a fragmentation step (Additional file 1). However, there is a sharp decline in reads mapping near the ends of the exons, indicating that reads do not overlap into introns leading to an accurate demarcation of exon boundaries. In contrast, the same analysis for tiling arrays produces more rounded curves with only a gradual drop at exon boundaries, signifying a poor exon boundary detection.

### Assessing Performance Relative to Annotation

**Figure 5:**
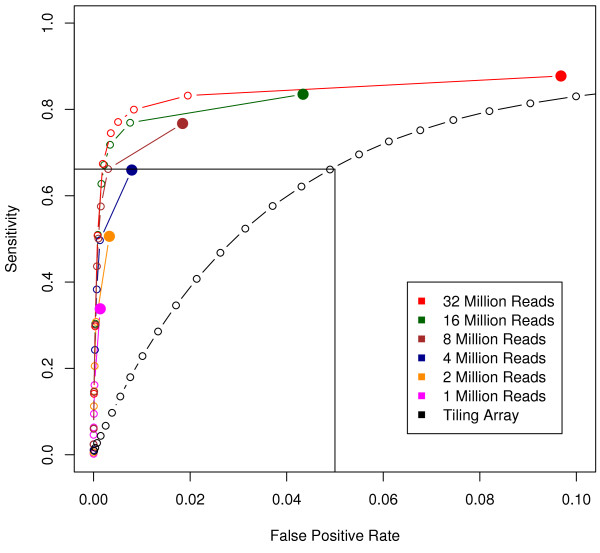
**ROC curve analysis**. Black: tiling array. Red: RNA-Seq with all 32 million reads. It is evident that the RNA-Seq substantially outperforms the tiling array with consistently higher sensitivity at lower FPR. Remaining curves are for RNA-Seq with only a subset of reads utilized. At an FPR = 0.05, just 4 million reads (blue) are required to attain the same sensitivity as two tiling array replicates.

In addition to exon boundaries, we assessed how accurate the two technologies are in predicting known transcribed and non-transcribed regions using ROC curves. The positives and negatives are taken from the gold standard set described previously. First, sets of TARs were generated for both the array and RNA-Seq data using the maxgap/minrun algorithm. Figure 5 depicts a ROC curve parameterized by signal threshold; this parameter affects specificity and sensitivity to a much greater extent than the maxgap and minrun. RNA-Seq performs substantially better; the area under the curve (AUC) is clearly larger than that of the array. For example, at a false positive rate (FPR) of 0.05, the tiling array yields a sensitivity of 0.68 while RNA-Seq attains a sensitivity of 0.85. This is consistent with previous results showing that expression levels between QPCR and RNA-Seq data are better correlated than with traditional microarrays or tiling arrays [9]. We found that the majority of TARs, 92%, overlap an exon while the remaining are in intergenic or intronic regions. Combined with the above result that tiling arrays have an average offset of 7 base pairs, we can conclude that much of the higher FPR of tiling arrays is due to its poorer detection of exon boundaries.

The red curve includes all 32 million mappable reads available for the L2-poly(A) sample. In addition, we asked how many reads are needed to achieve the same sensitivity as a microarray. We randomly selected subsets of the total reads to simulate the effect of limiting the depth of sequencing, and computed ROC curves as above for each of these. At an FPR of 0.05 for the array, we find that 4 million reads are required to achieve the same sensitivity as the two tiling array replicates. However, although the sensitivities are matched, the FPR of RNA-Seq is over five times better than that of the tiling array at this point.

### Cross-Hybridization

Thus far we have quantified the difference between the tiling microarray and RNA-Seq technologies. As expected, RNA-Seq consistently performs better by most measures. A major reason for this is likely due to the cross-hybridization that is a known issue in tiling arrays. Attempts to create predictive models of cross-hybridization [36] as well as empirically determined sequence based effects [37] have not led to general purpose methods for adjusting signal values to compensate for this issue. Thus, the best we can do is to understand the extent of cross-hybridization for the particular tiling array used in this study, which we did using pseudogenes and nearest neighbor probes. We also describe a method for determining the genomic regions that a particular array design does not interrogate reliably because of high sequence similarity.

#### Assessing Cross-Hybridization with Pseudogenes

Pseudogenes are known to arise in two ways. Processed pseudogenes result from the reverse transcription of mRNA back into the genomic DNA during retrotransposition. Since the pseudogene is derived from mRNA, it lacks a promoter region and is therefore usually not transcribed. Duplicated pseudogenes arise when a genomic region containing a gene is copied and a copy is subsequently disabled. In this case, the intron-exon structure is intact and the inactivity of the gene is due to sequence mutations [38,39]. We compiled a database of pseudogenes by running the Pseudopipe software [40], which provides a high confidence list of duplicated and processed pseudogenes and their respective parent genes. Importantly, a pseudogene and its parent have high sequence similarity but only the parent gene is likely to be expressed. Thus, high correlation between pseudogenes' expression levels and their respective parent genes' expression levels is suggestive of cross-hybridization, although there is evidence that a small fraction of pseudogenes are expressed [41].

Table 1a summarizes the results for duplicated pseudogenes for both technologies. For the tiling array, we find that 139 of the 258 duplicated pseudogene-parent gene pairs are not expressed. For duplicated pseudogenes, the hypothesis that a gene may be active but its pseudogene should not be is supported in 56 cases. However, in 40 cases the pseudogene is expressed at levels similar to its parent, and in 23 cases its expression is actually higher. Thus, in 63 cases, or about 25% of the total, we find evidence consistent with the cross-hybridization. In contrast, for sequencing only 8% (2 + 18 out of 258) of the pseudogenes exhibit expression levels similar to or higher than their parent genes. The results are similar for processed pseudogenes (Table 1b).

**Table 1:** Assessing cross-hybridization using pseudogenes

		Tiling Array					
		lower	equal	higher	non-expr	total	fraction
RNA-seq	lower	43	24	0	4	71	0.28
	equal	0	1	1	0	2	0.01
	higher	1	4	11	2	18	0.07
	non-expr	12	11	11	133	167	0.65
	total	56	40	23	139	258	1.00
	fraction	0.22	0.16	0.09	0.54	1.00	
		(a)					
		Tiling Array					
		lower	equal	higher	non-expr	total	fraction

RNA-seq	lower	28	12	1	4	45	0.21
	equal	0	2	0	0	2	0.01
	higher	0	3	6	0	9	0.04
	non-expr	8	36	12	100	156	0.74
	total	36	53	19	104	212	1.00
	fraction	0.17	0.25	0.09	0.49	1.00	
		(b)					

(a) The total row (column) gives the number of duplicated pseudogene-gene parent pairs from the tiling array (RNA-Seq) data where the relative expression level of the duplicated pseudogene is lower, equal, or higher than its parent gene. Non-expr means neither the pseudogene nor its parent are expressed. The equal and higher cases are indicative of cross-hybridization because the pseudogene, which is similar in sequence to its parent gene, is found to be expressed although most pseudogenes are believed not to be. The overlap between the two platforms is also shown for each combination of categories. For example, out of the 56 and 71 cases where the pseudogene's expression is lower than its parent gene for tiling array and RNA-Seq, respectively, 43 of the pseudogene-gene pairs are in common. (b) Similar results for processed pseudogenes.

#### Measuring Cross-Hybridization with Nearest Neighbor Classifiers

It has previously been demonstrated that cross-hybridization effects can be used to estimate transcription levels, even for TARs that are not specifically probed on a tiling array [42]. Motivated by this work, we considered the problem of predicting expression levels using probes that are similar in sequence to a given TAR but not within that TAR. A strong correlation between this predicted value and the actual intensity would suggest cross-hybridization is occurring.

**Figure 6:**
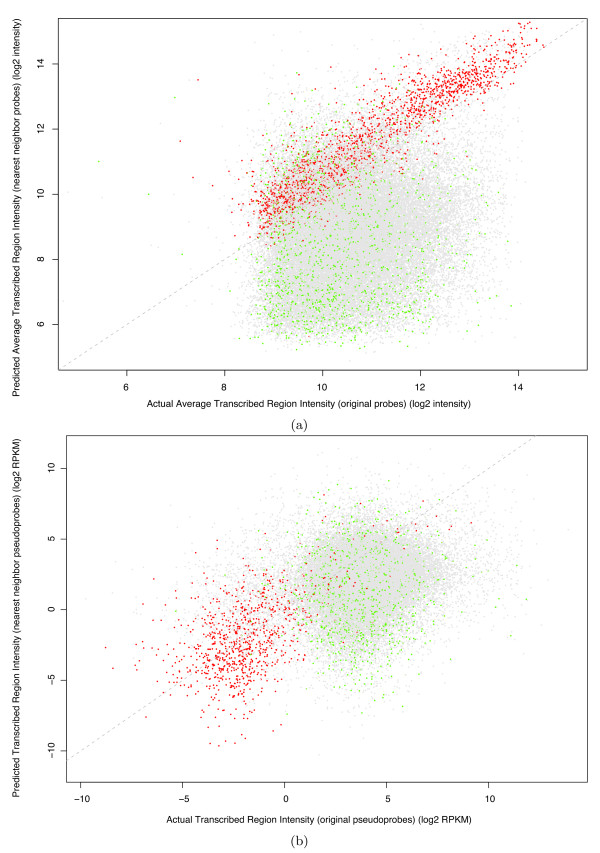
**Correlations between actual TAR intensities and that predicted by nearest neighbor probes**. TARs determined by tiling array data were tiled with virtual probes and assigned intensities using their nearest neighbors (see main text). Red points have an overall similarity score in the top fifth percentile (black list TARs; Additional file 2). Green points correspond to TARs having an overall similarity score in the bottom fifth percentile. Gray points are the rest. (a) Correlation between TAR intensities determined by the tiling array and the TAR intensities determined by using nearest neighbor probes. The intensities of TARs with high similarity to their nearest neighbor probes (red) are well correlated with the actual intensities (Spearman's correlation = 0.873). (b) Correlation between TAR intensities determined by RNA-Seq and the nearest neighbor "pseudoprobes." The correlation of highly similar TARs (red) is much lower (Spearman's correlation = 0.500).

We generated "virtual tiles" spanning TARs from our L2-poly(A) tiling array dataset. Briefly, virtual tiles are overlapping 25 bp subsequences of a TAR, each offset by 1 bp. Then, for each virtual tile, we found the probe with the highest similarity that didn't fall within its TAR--we call such a probe the nearest neighbor of the tile. To predict the intensity of a TAR, we simply averaged the intensities of the nearest neighbors. Figure 6a shows how the predicted and actual expression levels computed using tiling array data correlate for every TAR. It is evident that TARs with a high sequence similarity to their nearest neighbors correlate well (Spearman's correlation = 0.873), whereas the overall correlation is much lower (Spearman's correlation = 0.185). As further evidence, we used the pseudoarray to compute the correlation of the RNA-Seq intensity between the original pseudoprobes and their nearest neighbor pseudoprobes on the same set of TARs identified by tiling arrays. Here, TARs that are highly similar to their nearest neighbors have a lower correlation (Spearman's correlation = 0.500) than that of tiling arrays (Figure 6b). Moreover, according to RNA-Seq data, the expression of these high similarity TARs is significantly lower than the overall distribution (Wilcoxon rank sum, *p *< 2.2^-16^), which further supports the conclusion that they were incorrectly called expressed because of cross-hybridization.

**Figure 7:**
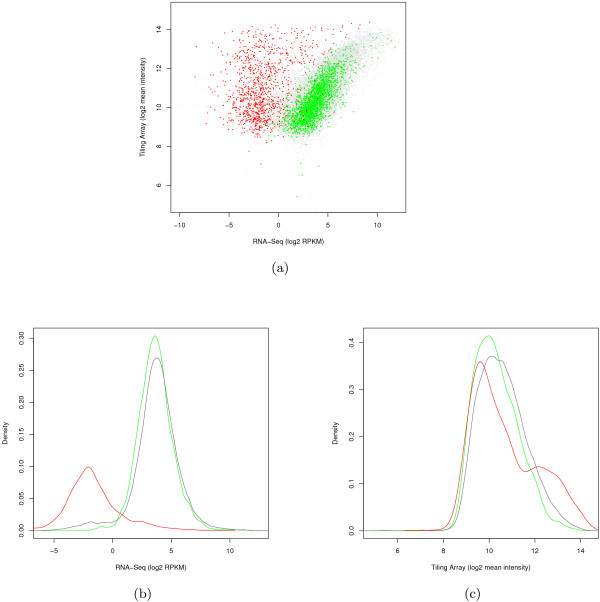
**Comparison of nearest neighbor analysis for tiling arrays and RNA-Seq**. (a) Correlation of TARs using intensities determined by RNA-Seq and tiling array. The colors scheme is identical to that in Figure 6a. As expected due to cross-hybridization, TARs with high similarity scores are called expressed by tiling arrays but not by RNA-Seq. (b) Density plot showing fraction of TARs (y-axis) with a given RNA-Seq expression level (x-axis). As above, TARs are segregated by similarity. It is clear that TARs with the highest similarity (red) fall in a different distribution and are generally not expressed according to the RNA-Seq data. (c) Similar but x-axis is the TAR intensity from tiling arrays. In this case, highly similar TARs are more likely to be highly expressed, suggestive of cross-hybridization. The distribution of highly similar TARs exhibits a bump, possibly due to more highly expressed TARs being more likely to exhibit cross-hybridization.

We then created density plots of the expression levels measured by RNA-Seq and tiling arrays (Figure 7). It is apparent that high similarity regions do not fall into the overall distribution in RNA-Seq. Strikingly, these regions are expressed at low levels when measured by RNA-Seq, but highly expressed when measured with tiling arrays. This is exactly the pattern we would expect from cross-hybridization. We collected this set of high similarity TARs into a master list of "black list" regions whose probing by the tiling array is potentially unreliable (Additional file 2). The list includes 2,327 regions covering a little over half a percent of the genome.

### Utilizing RNA-Seq to Calibrate Tiling Array Data

Some of the analyses we have described earlier required us to segment the tiling array signals into TARs. Here, we describe our method for doing this, which consists of searching amongst possible combinations of the algorithm's parameters to pick optimal ones. Then, we will describe a method for assigning each TAR a rank score by comparing them to null regions of the annotation, and also assign each TAR a "marginal FPR." These first steps are applicable to all arrays, not just those with matched RNA-Seq data. Then, for arrays with matched RNA-Seq data, we describe a technique for adjusting the marginal FPR by using the RNA-Seq data as the gold standard set instead of the annotation. This is expected to improve the results because the RNA samples are matched, whereas the WormBase annotation is not specific to the sample under consideration.

#### Optimal Segmentation Algorithm

First, the tiling array signals are segmented into TARs using the maxgap/minrun algorithm. Briefly, a contiguous sequence of probes exceeding the signal threshold *T *is joined together to form a TAR. A number of base pairs are allowed to fall below the threshold within a single TAR--this parameter is the maxgap *G*, and regions shorter than some minimum length are excluded--known as the minrun *R*. This approach can be readily applied to segment RNA-Seq data also.

**Figure 8:**
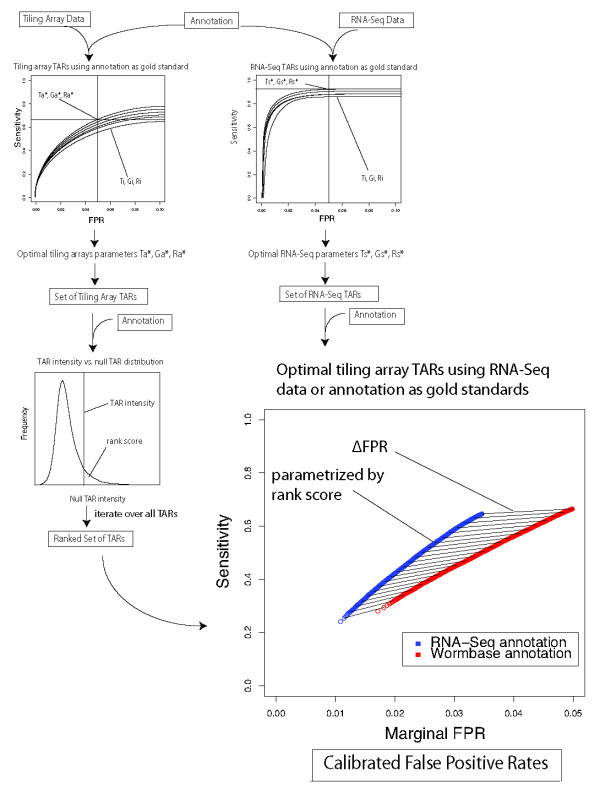
**Schematic describing the tiling array analysis and FPR calibration pipeline**. First, we optimize the threshold, maxgap, and minrun parameters of tiling arrays and RNA-Seq segmentation, notated T, G, and R, respectively. To do this, we compare the called TARs to a manually curated gold standard set and do a brute-force search over the parameter space to attain an FPR of 0.05 with maximum sensitivity. Then, as detailed in the main text, we calculate a rank score for each tiling array TAR by comparing its intensity to a distribution of null TARs constructed from non-exonic regions. We then map this value to a marginal FPR, which is calculated by sorting the TARs based on their rank score and then iteratively selecting smaller subsets of TARs, assigning the FPR to the TAR defining the outermost boundary. This marginal FPR can then be adjusted by following a similar procedure using the RNA-Seq data as a gold standard set, giving a calibrated marginal FPR for each TAR.

One of the main challenges in effectively employing this algorithm is selection of the signal threshold, maxgap, and minrun. We addressed this by using a brute-force approach to find optimal choices for these parameters. We selected a range of physically reasonable values for each parameter, and computed the set of TARs for each of a large combination of values within these ranges. Then, for each set of TARs, we computed the sensitivity and FPR against the gold standard set of positives and negatives (Figure 8). We defined the optimal choice of parameters as those maximizing the sensitivity at an FPR of 0.05, and implemented an algorithm to automatically determine these parameters within a small tolerance. This gives an optimal segmentation of the signal, which we used in our analyses. The optimal threshold, maxgap, and minrun are notated , and  for arrays and , and  for sequencing.

#### Rank Score and Marginal FPR Calculation

Next, we assigned a score to each TAR that ranks the TARs in order by likelihood of expression. First, we constructed a null distribution of probes that are contained in regions not annotated as exonic. Then, for a given TAR containing a certain number of probes, we generated a large number of regions of equal length from the null set of probes. The rank score is defined as the fraction of these null regions whose mean intensity exceeds that of the TAR in question (Methods). Thus, a smaller rank score represents greater confidence that the given TAR is expressed.

The rank score is informative, but it is also helpful to map this into a "marginal FPR," which has a more concrete interpretation. The marginal FPR represents the FPR that would be obtained if the TAR in question is the least confident TAR retained. In other words, given a list of TARs ordered by their rank score, one can easily choose the subset of TARs that would give a desired FPR. To calculate this, the TARs are ranked by rank score from largest (least confident) to smallest (most confident). We then iteratively consider subsets of these TARs by setting an increasingly stringent rank score cutoff. For each such subset, we compute an FPR by comparing it to the annotation, and call this the marginal FPR of the least confident TAR still in the set (Methods).

#### Adjusting the Marginal FPR Based on RNA-Seq

The analysis method up to this point does not require a matched RNA-Seq data set. We now consider a method for refining the marginal FPR based on the case that matched sequencing data is available. The first step is to segment the RNA-Seq data as described previously, and then use these sequencing TARs as gold standard positives and the remaining regions as gold standard negatives. Then for each array TAR, we compute the FPR, now using the sequencing based set of positive and negative regions. Figure 8 shows that the ROC curve is improved with regard to both sensitivity and FPR. Furthermore, the lines between the ROC curves connect points corresponding to the same subset of array TARs. Thus, we can see how much the marginal FPR shifts and call this the ΔFPR.

Note that the particular ΔFPR is different for each subset of TARs, each of which corresponds to a particular rank score. We write ΔFPR(r) to indicate ΔFPR's dependence on r. This serves as a calibration of the original marginal FPRs, which is adjusted to FPR + ΔFPR(r) (Methods).

In this particular case, the ROC curves are reasonably close to each other and the ΔFPRs are small. This indicates that assigning FPRs based on the conservative annotation we have been using is reasonable, but in other cases the calibration could be used to refine the analysis.

## Discussion

We demonstrated that most gene expression levels are well correlated between RNA-Seq and tiling arrays. There are some outliers, which are generally called highly expressed by tiling array and poorly expressed by RNA-Seq. In previous studies, similar outliers were also found and when analyzed with qPCR, it was evident that their profile was masked by cross-hybridization [23]. To further bolster this conclusion, we note that a substantially greater number of inactive pseudogenes are called expressed by tiling arrays (Table 1) when compared with their paralogous parent genes. Furthermore, TARs tiled by probes that are highly similar to their nearest neighbors also tend to be called expressed by tiling arrays and not by RNA-Seq, strong evidence of cross-hybridization.

We have demonstrated that a simple similarity score threshold for tiling array probes can identify potentially unreliable regions (Figures 6a, 7). To immediately aid researchers conducting tiling array analysis on *C. elegans*, we provide our manually compiled list of such "black list" regions (Additional file 2). It is important to note, however, that these unreliable regions are dependent on the design of the tiling array and possibly on other factors such as hybridization conditions; an analysis like ours would need to be re-run in other scenarios.

Besides cross-hybridization, another drawback of tiling arrays is the limited dynamic range of detection [9]. Previous work has presented RNA-Seq data with a dynamic range varying over 5 orders of magnitude [8]. Consistent with this, we note that ~40% more genes are called differentially expressed by RNA-Seq between two distinct subpopulations of *C. elegans*, even when using a conservative statistical test. It is also clear that the fold difference of differential expression is greater for RNA-Seq (Figure 2a). Bloom et al. deliberately used fewer reads and a high number of array replicates in their comparison of differential expression in *S. cerevisiae *[43]. This defines "fair comparison" in an alternative way, one where the cost of experimentation is similar, and they find that arrays better distinguish differential expression for low abundance transcripts. This is because those transcripts' specific probes will still exhibit hybridization while the few reads may not be picked up in sequencing.

Yet another drawback of tiling arrays is the comparative lack of exon boundary resolution. Not unexpectedly, the median absolute deviation of aggregated exon boundaries is much smaller for RNA-Seq than for tiling arrays, reflecting the size of the oligonucleotide probes in our tiling arrays. This distinct difference between the two technologies is especially important when sequencing unannotated transcriptomes and detecting alternative splicing; these results accentuate why RNA-Seq has been so successful at both types of analysis [15,18,44].

Given the superiority of RNA-Seq using these metrics, our strategy of using RNA-Seq as a gold standard set for guiding tiling array analysis may be useful for calibrating experiments where large numbers of tiling array runs are required. It is conceivable that one or two "pilot" RNA-Seq experiments could guide a series of microarrays. Indeed, a variant of this strategy was used successfully when validating the *de novo *assembly of the Glanville fritillary butterfly's transcriptome [45]. It may also prove useful for probing the transcriptomes of organisms with poor transcriptome annotation. This general strategy has the potential to be expanded from the maxgap/minrun algorithm to other methods, such as hidden Markov segmentation. We find that the false positive rate determined for tiling array TARs decreases by an average of ~10% when using the RNA-Seq data as a gold standard set instead of the high-confidence WormBase annotation. Even though using the annotation as a gold standard set is not optimal, because not all annotated transcripts are necessarily expressed, it is satisfying to observe that the effect is relatively small. Given the wealth of expression data coming from both RNA-Seq and tiling array analyses, it is often difficult to understand how to interpret cross-platform results. As a first step, we examined the relationship between transcriptome coverage and quality of called TARs. Furthermore, we determined the approximate number of reads required to yield a sensitivity comparable to that of tiling arrays at a given FPR. This is of practical importance to researchers employing RNA-Seq, since the cost of sequencing is generally proportional to the number of reads obtained. It is important to note, however, that the transcriptome is dynamic--expression can vary widely between different life stages and growth environments. For the L2-poly(A) *C. elegans *transcriptome, we find that 4 million reads are necessary to achieve a similar sensitivity to tiling arrays. Importantly, because of its single nucleotide resolution, the FPR of RNA-Seq at this sequencing depth is >5x greater than that of tiling arrays.

In order to extend this conclusion to other organisms, we outline a simple method of approximating transcriptome coverage. In principle, the coverage of the transcriptome could be calculated if we knew the exact number of base pairs of RNA present at a given point in time. Since this is difficult to measure, we can approximate this number for organisms whose transcripts are well annotated, by assuming that the total number of base pairs of RNA in the cell is proportional to the total number of base pairs of annotated transcripts by some constant *c*. This approximation makes the assumption that varying transcript expression levels averages out across the transcriptome. Thus,

where *L *is the number of annotated exon base pairs, including isoforms to account for complexity of transcription, *N *is the total number of reads within annotated exons, and *R *is the average read length. It is reasonable to assume that *c *should be relatively constant across organisms, and so this coverage value may be meaningful for organisms other than *C. elegans*.

Although almost all of our analyses have indicated otherwise, there are some drawbacks for RNA-Seq. "Cross-mapping" is an analogous problem to cross-hybridization, and has been addressed in complex organisms [8] particularly because it poses a problem for genomes with many repetitive regions. We included only high quality mapped reads, but allowing greater mismatches, which could be beneficial for detecting additional transcription, would lead to decreased confidence in the transcriptional activity of regions with high sequence similarity. Our analysis was less affected by this issue since the *C. elegans *genome does not contain many repetitive regions (~87% is non-repetitive; [46]), and also because we included a rigorous pre-processing step that left less than 3% of reads mapping to multiple locations in the *C. elegans *genome [13]. In principle, however, it is important for users of RNA-Seq to consider the repetitiveness of the genome they are analyzing, to determine how many reads map to multiple locations, and understand how they are dealt with. Importantly, different software packages deal with ambiguous reads differently; for example, MAQ assigns these reads randomly, whereas cross_match gives information about the alternative mapping sites. Ironically, it is evident that tiling arrays are not immune to the problem of ambiguous mapping; indeed ~6% of tiling array probes used in this study map to multiple locations in the *C. elegans *genome (Additional file 1: Table S2).

As read lengths continue to get longer, however, the problem of ambiguous read mapping will certainly become less of an obstacle. Indeed, the tantalizing possibility of obtaining kilobase long reads may completely eliminate this altogether. However, it has recently been demonstrated that transcript length affects differential expression analysis [47]. Furthermore, the problem of rRNA and tRNA overloading the reads often forces RNA-Seq users to purify RNA over a poly-dT column, potentially losing RNA species of interest. This problem is currently being bypassed with the increased availability of kits for specific removal of rRNA from total RNA samples (Ambion, Invitrogen).

A less tangible disadvantage of RNA-Seq is the requirement for "big data," which can cause problems in storage, portability, and processing time [48]. For example, just the sequences from the L2-poly(A) RNA-Seq dataset take up ~13 gigabytes. For genomes larger than *C. elegans*, which require more reads, this number can rapidly increase. Larger data is simultaneously more costly to archive and easier to corrupt. Furthermore, these large datasets can often strain computational resources with respect to both processing time and memory usage. Although great strides have been made, as RNA-Seq grows in popularity, it is imperative that highly efficient RNA-Seq software pipelines and data formats be developed.

## Conclusions

We compared the relative merits of tiling arrays and RNA-Seq by investigating the transcriptome of a matched *C. elegans *sample. Both platforms effectively detected transcript expression levels and their raw signals were highly correlated. RNA-Seq however finds a greater number of differentially expressed genes and excels at accurately detecting exon boundaries. As technical obstacles are overcome and highly efficient software pipelines are constructed for RNA-Seq, its increased specificity and sensitivity will undoubtably be a major boon for transcriptomics. Its resolution of exon boundaries and ability to detect alternative splice variants is unparalleled. In addition RNA-Seq data contains actual sequence information that can be used for applications like SNP detection that cannot be identified from tiling array data. On the other hand, tiling arrays remain cost effective for many species and perform reasonably well with respect to expression levels, with the caveat of cross-hybridization effects. It will be important to continue investigating the relative merits of these technologies and to carefully select the appropriate platform based on the biological questions being addressed.

## Methods

### Correlations

For each base pair in the WS170 build of the *C. elegans *genome, the reads mapping to the plus and minus strand of that base pair were added together to give an overall score. Then, for each gene in the composite model, the RPKM was calculated as in [8] for the L2-poly(A) RNA-Seq data. To calculate the intensity per composite gene from the tiling array analysis, a probe was considered to be within a composite exon if it was wholly enclosed by that exon. Then, the average probe intensity from the smoothed L2-poly(A) data was assigned to that gene.

### Differential Expression

In order to make a fair comparison between tiling arrays and RNA-Seq, "pseudoarrays" were constructed by calculating an RPKM using either the L2-poly(A) or YA-poly(A) RNA-Seq data for each perfect match probe on the tiling arrays. When used in conjunction with the WormBase composite gene model, the pseudoarray predicts gene expression levels almost identically with the raw sequencing data (Spearman's correlation = 0.99, Additional file 1: Figure S1). This pseudoarray was then treated identically as its tiling array counterpart for the rest of the analysis.

For the tiling array, we used L2-tot and YA-tot RNA samples. We did not employ the L2-poly(A) array data for this analysis to avoid skewing the calculation against YA-tot, which was the only young adult data available for the array. This should not affect the results substantially since we found that both L2-tot and L2-poly(A) correlate reasonably well with the RNA-Seq L2-poly(A) data (Figure 1; Additional file 1: Figure S4).

To determine differential expression for both technologies, their respective YA and L2 data were quantile normalized as in [49]. As before, a probe or pseudoprobe was assigned to a composite gene if it fell wholly within a composite exon. We then used the Wilcoxon rank sum test to compute a p-value for each composite gene's set of probes between the YA and L2 datasets. The p-values were then transformed to q-values using the method of [34]. If a gene's q-value fell below 0.01, it was considered differentially expressed.

### Exon Boundary Detection

To assess the accuracy of exon boundary detection we selected a set of TARs from both the tiling arrays and RNA-Seq (Additional file 1). Next, for every exon in the gold standard set, we determined the overlap between its boundaries and the corresponding boundary of an overlapping TAR, if any. The offset is defined as positive or negative if the TAR boundary extends beyond or falls short, respectively, of the exon boundary. TARs that overlapped with more than one annotated exon were excluded. Lastly, the offsets from both the 5' and 3' exon boundaries were collected and the offset distribution was plotted. We used the gold standard set from [13].

### Pseudogene Analysis

Worm pseudogenes were obtained from pseudopipe [40], which is itself based on the WS170 build of the *C. elegans *genome. A total of 530 duplicated pseudogenes and 257 processed pseudogenes were found. The genomic coordinates of the associated parent genes were obtained using Ensembl. After setting a further requirement that there must be at least six array probes in the pseudogene and its parent gene, we obtained 258 duplicated pseudogenes and 212 processed pseudogenes.

Using the matched L2-poly(A) samples, a pseudogene was called expressed if it passed a minimum array intensity threshold of 100 or minimum read count of 1. We added noise from a normal distribution centered about zero with a standard deviation of 0.1 to pseudogene and parent gene values to prevent ties. If both the pseudogene and parent gene were called expressed, the Wilcoxon rank sum test was utilized to determine if the expression level of the pseudogene is "higher," "lower," or "equal" to its parent gene, using a p-value cutoff of 0.01. If the pseudogene or parent gene were both not expressed, they were considered equal. If one was expressed and the other wasn't, they were considered differentially expressed.

### Nearest Neighbor Analysis

Once we determined the TARs from the tiling arrays, we constructed a set of "virtual tiles" for each TAR. We tiled the TARs with 25 bp probes with an offset of 1 bp. This resulted in about 21 M tiles covering all the TARs. For each virtual tile, we then searched for its nearest neighbor probe, i.e. the tiling array probe with the most similar sequence. We searched in the database composed by about 6 M probes, since we considered perfect-match and mismatch probes independently. To do this, we employed blat [50] with parameters *tileSize *and *minScore *set to 8 and 12, respectively, in order to adjust for the short reads. Each virtual tile may have one or more probes with different levels of similarity. We chose the most similar, i.e. the one with more nucleotides in common, but we excluded probes that are located within the same TAR to ensure we obtain an accurate estimation of the cross-hybridization signal. We then assigned to each virtual tile the intensity of its nearest probe. We finally estimated the expression level of the nearest neighbor TAR by computing the average intensities of the virtual tiles and compared this value with the expression level measured from the actual probes within the TAR (determined by averaging their PM-MM values). For each TAR, we determined the similarity score by taking the mean of the similarity of all its nearest neighbor probes.

Finally, each TAR is characterized by its intensity value measured by the tiling probes and the RNA-Seq, the intensity values and average similarity estimated from its nearest neighbor probes. To create our master list of cross-hybridization TARs, we selected those in the top 5% similarity score (highly similar TARs). This threshold was selected to yield a reasonable separation of intensities between the highly similar TARs and the overall TARs.

### Tiling Array Rank Score Calculation

For each TAR called by tiling arrays, we computed a rank score for every TAR to give an estimate of how likely it is that the TAR is truly transcribed. As a first step, we created a null distribution of probes that are likely not transcribed. We considered any probe that did not fall into an exon marked as "confirmed" by WormBase to be in the null distribution. This liberal choice of a null distribution allowed us to create the rank scores with higher resolution.

For each TAR, we determined the number of probes *L *falling within its boundaries, and created a set of 500,000 null TARs, each also having *L *probes, by selecting null probes at random. Then, the rank score is simply A/500000, where *A *= the number of intergenic TARs whose mean intensity is above that of the TAR in question. It is important to note that the lower the rank score, the more confident we are that the TAR is expressed. Furthermore, this value is not monotonic with intensity, since it is dependent on the TAR length.

### Marginal FPR Calculation and Adjustment

Let [TAR_1_,TAR_2_, ..., TAR_*N*_] represent the list of *N *TARs in descending order by rank, so that the first TAR is the least confident one. Then, we compute the FPR against the annotation for this entire list, and call this the marginal FPR of TAR_1_. Next, we remove TAR_1_, compute the FPR for the list [TAR_2_, ..., TAR_*N*_], and assign this as the marginal FPR of TAR_2_. Then, TAR_2 _is removed and the process is continued for each subset of TARs.

The FPRs above are computed using the annotation. This procedure is then repeated, except using the matched RNA-Seq TARs as the gold standard set, leading to an alternative marginal FPR for each TAR. The difference between the two marginal FPRs is notated ΔFPR and can be used as an adjustment for TARs with the corresponding rank score.

## Authors' contributions

AA and DK led the analysis and prepared the bulk of the manuscript. AS and LH performed the nearest-neighbor and exon boundary analyses. AS and JR designed the rank score and marginal FPR calculations. RS assisted in defining the TAR calling and differential expression algorithms as well as generating the pseudogene set. LWH and RHW led the RNA-Seq experimental efforts, and LWH conducted the analysis to provide RNA-Seq signals. VR led the tiling array experimental efforts. JR, RHW, and MG oversaw the project and provided general guidance. All authors reviewed the manuscript.

## Supplementary Material

Additional file 1 Supplementary material
Detailed descriptions of experimental and analysis methods, and additional results. Click here for file

Additional file 2 **Black list regions**. List of regions deemed unreliable, for the tiling array used in this work, based on nearest neighbor analysis. Click here for file
