# Supplementary material for: Comparison and calibration of transcriptome data from RNA-Seq and tiling arrays
Source: BMC Genomics. 2010 Jun 17;11:383. doi: 10.1186/1471-2164-11-383 (PMC3091629; doi:10.1186/1471-2164-11-383)
Supplement: Additional file 1 — Supplementary material Detailed descriptions of experimental and analysis methods, and additional results. [file 1471-2164-11-383-S1.PDF]

Supplementary Material
for
Comparison and calibration of transcriptome data from RNA-Seq and tiling arrays

1 Sample Preparation

Animals were synchronized by treating a mixed stage hermaphrodite population with bleach to collect embryos. The embryos surviving bleach treatment were incubated without food, causing the larvae to arrest at the L1 stage upon hatching. Starved L1 larvae were plated on standard NGM plates in the presence of food (OP50) and raised at 25°C. Staging was determined by examining a subset of animals under Nomarski optics at intervals for vulval and germline development.

To isolate RNA, we first centrifuged medium- to large-scale preparations of staged *C. elegans* nematodes on a sucrose cushion to remove debris. The worms were then recovered in several washes of S basal. Total RNA isolation was performed by adding 4 volumes of Trizol (Gibco) per volume of packed worms. After two rounds of freeze/thaw and vortexing, the slurry was extracted with 2 volumes of chloroform. The aqueous layer was precipitated with one volume of isopropanol and the pellet washed with 70% ethanol. The pellet was resuspended in water and the concentration determined by UV spectrometry. RNA quality was assessed by examining an aliquot run on an ethidium bromide stained gel for discrete ribosomal and transfer RNA bands, as well as an mRNA "smear".

2 Tiling Array

We followed the standard Affymetrix protocol for cDNA synthesis, using the GeneChip®WT Double-Stranded cDNA Synthesis Kit (#900813). The cDNA was then labeled using reagents from Affymetrix. 7.5 μg double stranded cDNA is combined with 4.8 μl of 10X Fragmentation buffer, 1.5 μl of UDG (10U/μl), 2.25 μl of APE 1 (100U/μl), and H_20 to 48 μl. The samples were incubated at 37°C for 1h, 93°C for 2 min, and 4°C for 2-10 min. Of this reaction, 45 μl was placed in a new tube. In a separate tube, 12 μl of 5X TdT buffer, 2 μl of TdT (30U/μl), and 1 μl of DNA labeling reagent (5mM) were mixed together. This mixture was then added to the 45 μl of fragmented dsDNA, mixed, and incubated at 37°C for 1h, 70°C for 10 min, and 4°C for 2-10 min. Array hybridization and processing were performed at the Keck Facility at Yale University using standard Affymetrix protocols for tiling array hybridization.

Affymetrix's GCOS software was used to convert the tiling array hybridization images to numerical values indicating the intensity of each perfect-match (PM) and mismatch (MM) probe. These are provided as CEL files for each tiling array replicate. The L2-poly(A) dataset had 2 tiling array replicates; the rest had 3 replicates. Affymetrix also provides an array design file that specifies the location of each PM probe on the *C. elegans* genome, based on the WormBase WS170 build. We combined the CEL files with this BPMAP file to map the intensity values on to the genomic coordinate, assigning each interval the PM - MM value. A pseudomedian smoothing filter was applied with a window size of 110 base

Pseudo-replicate 1 (num reads)	Pseudo-replicate 2 (num reads)	Correlation
1M	2M	0.980
2M	4M	0.990
4M	8M	0.995
8M	16M	0.991
16M	32M	0.998

Table 1: Correlations between RNA-Seq pseudo-replicates. Pseudo-replicates were constructed by randomly selecting various numbers of reads from all available. Pearson correlations were then computed for the given pairs of resulting signals.

pairs prior to segmentation [4]. We then used the maxgap/minrun algorithm to segment the tiling array data into TARs [5, 3]. The selection of optimal parameters is discussed in the main text. Based on a sensitivity analysis, we found the maxgap and minrun to have minimal effect on the results within a reasonable range. For L2-polyA, our final parameters were $T_a^* = 556$, $G_a^* = 30$ bp, and $R_a^* = 100$ bp, giving an FPR of 0.050 and a sensitivity of 0.67.

3 RNA-Seq

The same samples as described above for the tiling array were sequenced. The RNA-Seq data was derived from [2] and details are available in the supplement associated with that publication. Briefly, the total RNA was then prepared for sequencing by size-fractionating the polyA+ RNA and excising the 100 to 300 bp fractions. Gel-purified cDNA was modified using the Illumina genomic DNA sequencing kit, and after sequencing with the Illumina 1G analyzer, the Firecrest and Bustard applications were run with default parameters to obtain reads from the image files. The resulting reads were then aligned to the genome using MAQ, and to a splice junction and splice leader database using cross_match. Results from these alignments were combined to create an overall coverage count per base pair. Only reads that uniquely mapped were used in this analysis.

We observed the RNA-Seq data to have very high signal-to-noise and thus did not obtain replicates. We confirmed this by constructing pseudo-replicates. We randomly selected 1M, 2M, 4M, 8M, 16M, and all 32M of the total read set. We then computed the Pearson correlations between pairs of the resulting signals. Table 1 shows that all correlations are very high (≥ 0.98).

Maxgap/minrun segmentation was carried out by combining the coverage count of the positive and negative strand of each base pair, and then optimizing parameters as described in the text. The final parameters chosen for sequencing the L2 life stage were $T_s^* = 2$, $G_s^* = 5$ bp, and $R_s^* = 50$ bp, giving an FPR of 0.020 and a sensitivity of 0.83.

The sequencing data has base pair resolution, while the array data provides a signal value for every 25 bp probe. In some of our analyses, it was necessary to construct a "pseudoarray" from the sequencing data to put it on the same basis as the tiling array. We did this by computing the RPKM over all read counts for the coordinates within the array probes. Figure 1 shows that the signal properties are not significantly altered by this transformation.

4 Gold Standard Annotation

All analyses presented were based on the WormBase WS170 genome build and the corresponding WS170 annotation [6]. WormBase provides a comprehensive annotation that includes regions confirmed with EST evidence and also those predicted from various gene finding programs. We use a conservative subset of this annotation as defined by [2]. Briefly, only introns identified as "Confirmed" are included and any that are identified as a coding gene or a pseudogene by any gene prediction program are excluded. Only those exons

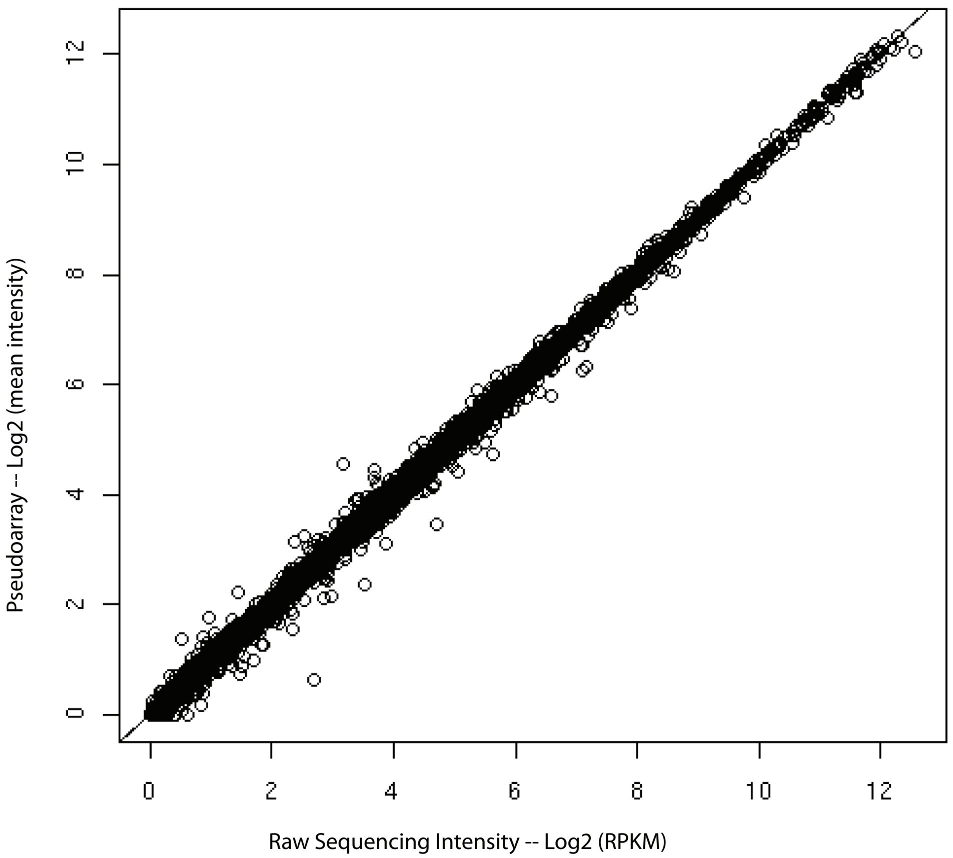

Figure 1: Raw sequencing vs. pseudoarray gene expression levels using the WormBase composite gene model. The pseudoarray performs almost identically with the raw sequencing (Spearman's correlation = 0.99, Adjusted R-squared = 0.999) when a large enough number of data points are included (i.e., when binned into genes instead of just exons).

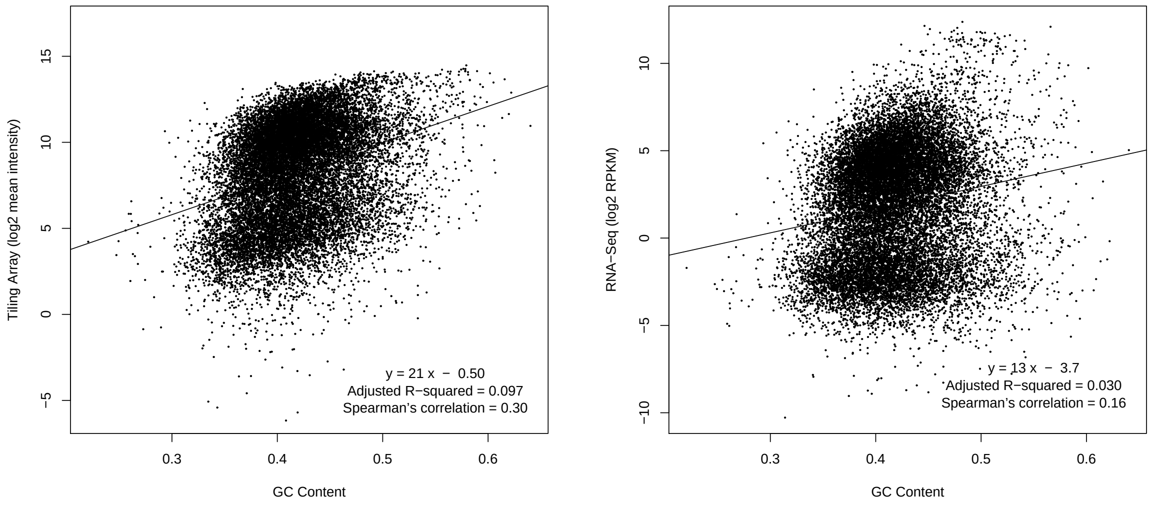

(a) (b)

Figure 2: GC Content vs. Expression Levels using PolyA-enriched L2 samples. (a) GC Content vs. Tiling Array Gene Expression Levels. GC content is significantly correlated with gene expression levels (Spearman's correlation = 0.30; Kolmogorov-Smirnov test, $p < 10^{-15}$, D = 0.1522). (b) GC Content vs. RNA-Seq Gene Expression Levels. Interestingly, GC content is still significantly correlated with gene expression levels using RNA-Seq data, albeit to a lesser degree (Spearman's correlation = 0.16; Kolmogorov-Smirnov test, $p < 10^{-15}$, D = 0.0991).

flanked by Confirmed introns are included and only those not called intronic by any program. Intergenic regions are those not annotated as pseudogene, RNA, exonic, or intronic, and also bases within 200 bp of the start of a CDS for within 700 bp of the 3' end of a CDS are excluded.

For the ROC curve analyses, exonic nucleotides were considered positives and both intronic and intergenic nucleotides were taken as the negatives. Sensitivity and false positive rates were then calculated as described in the literature [1, 7].

For analyses relying on a single gold standard set of exons, we must decide how to treat alternative isoforms to avoid double counting exons. We handled this by constructing a "composite gene model" by projecting isoforms onto the genome, in effect taking the union of all annotated exons. We call the resulting exons "composite exons."

5 GC Content

RPKM and mean intensity were calculated as described above. The WS170 build of the *C. elegans* genome was used to infer the GC content for each composite gene, excluding introns as usual. In order to determine the significance level of the bias, we first set an expression threshold of the median of the RNA-Seq or tiling array data, respectively, and compared the resulting GC distribution to the overall GC distributions with the Kolmogorov-Smirnov test.

6 Mapping Bias

In order to determine whether the TARs predominantly overlap with the 5' or 3' end of annotated exons, the TARs were intersected with the exon annotation and aggregated over all exons (Figures 3a, 3b). The exon length was normalized from zero to one in order to

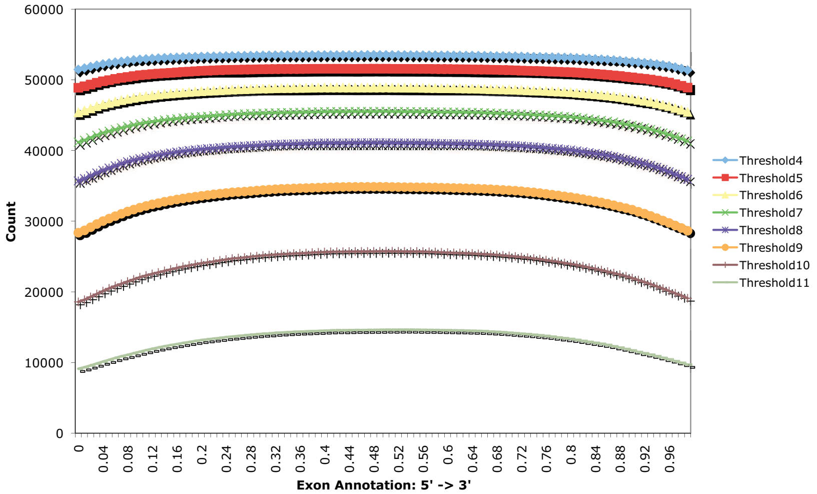

(a)

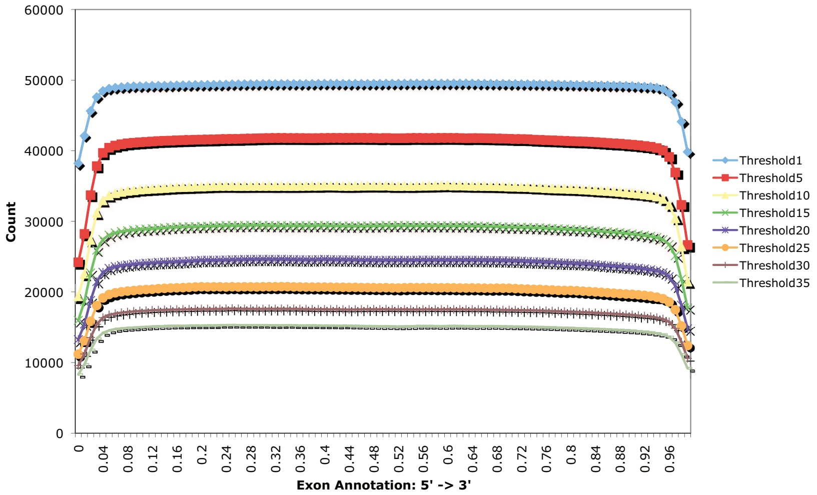

(b)

Figure 3: Aggregated Mapping Bias of RNA-Seq and Tiling Array. The set of TARs was intersected with exons, and the relative location from the 5' to 3' end where the TARs intersect were aggregated over all exons. The x-axis is the normalized location along exons, and the y-axis is the number of TARs that overlapped at that location. The analysis was conducted for TARs computed for a range of thresholds. The symmetrical shape of the curves for both (a) tiling arrays and (b) RNA-Seq indicate that there is no mapping bias in both platforms. However, the gradual curves in the array indicate that array TAR boundaries do not coincide will with exon boundaries. In contrast, the flat curves with sharp drops at boundaries for RNA-Seq indicate that RNA-Seq TAR boundaries coincide closely with exon boundaries.

Perfect Matches	Probes
**0**	**4,160 (control probes)**
**1**	**2,942,368**
**≥2**	**182,081**
2-5	141,976
6-10	16,147
11-20	12,556
≥21	11,402
**total**	**3,128,609**

Table 2: Mappability of array probes.

account for various exon lengths. Zero represented the 5' end of an annotated exon while one denoted the 3' end of an exon. These normalized coordinates were also adjusted if the annotated exon was on the negative strand. Subsequently, the number of TARs was plotted on the y-axis as a function of exon annotation in the 5' to 3' direction. We used the gold standard set described above.

7 Probe Mappability

We determined the mappability of probes by aligning them against the *C. elegans* genome (WS170). We employed eland_25, a short-read alignment tool from the Illumina Genome Analyzer Suite. The probes are expected to map to a unique location in the genome. However, we found that ~6% of the probes can be mapped to multiple locations. Table 2 reports the numbers.

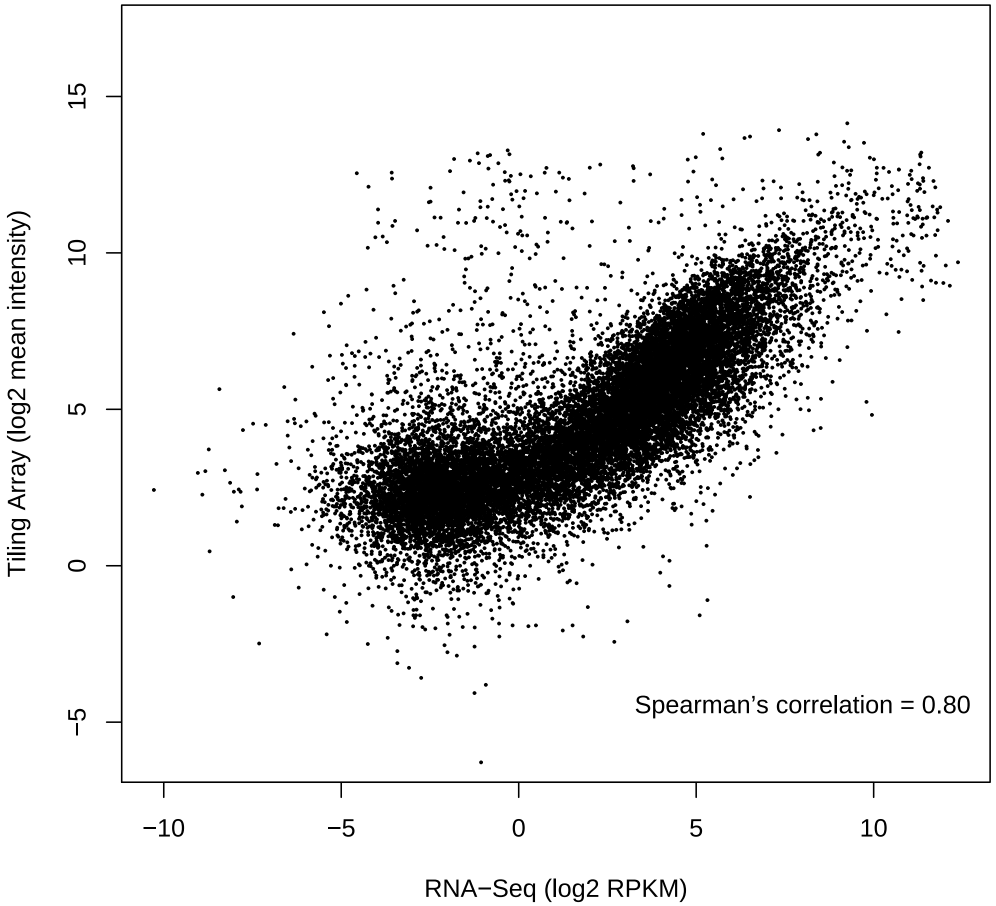

Figure 4: As in main paper Figure 1, except that the tiling array is hybridized with L2-tot RNA. The expression levels are still reasonably correlated although less so with a Spearman's coefficient of 0.80.

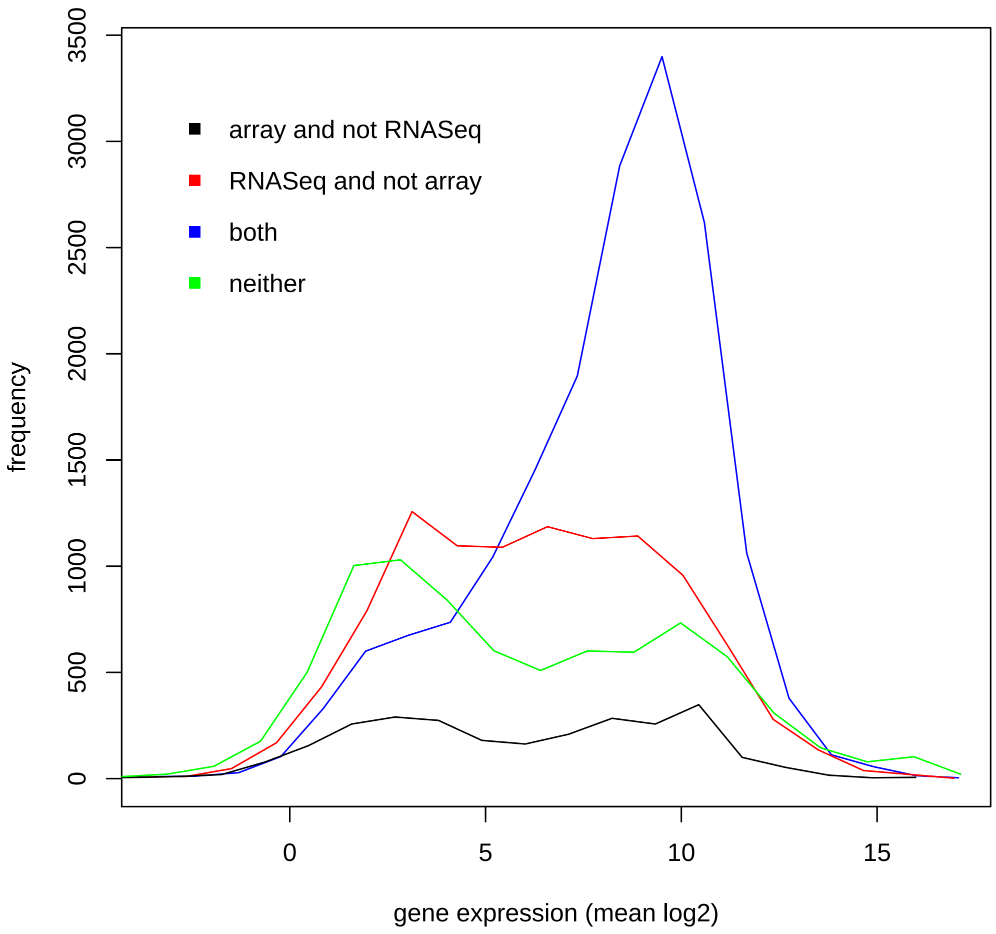

Figure 5: As in main paper Figure 3, but gene expression levels are based on RNA-Seq data instead of tiling array data.

References

[1] Tom Fawcett. An introduction to ROC analysis. *Pattern Recogn. Lett.*, 27(8):861–874, 2006.

[2] Ladeana W Hillier, Valerie Reinke, Philip Green, Martin Hirst, Marco A Marra, and Robert H Waterston. Massively parallel sequencing of the polyadenylated transcriptome of *C. elegans*. *Genome Research*, 19(4):657–66, April 2009.

[3] Dione Kampa, Jill Cheng, Philipp Kapranov, Mark Yamanaka, Shane Brubaker, Simon Cawley, Jorg Drenkow, Antonio Piccolboni, Stefan Bekiranov, Gregg Helt, Hari Tammana, and Thomas R Gingeras. Novel RNAs identified from an in-depth analysis of the transcriptome of human chromosomes 21 and 22. *Genome Research*, 14(3):331–342, March 2004. PMID: 14993201.

[4] Thomas Royce, Nicholas Carriero, and Mark Gerstein. An efficient pseudomedian filter for tiling microrrays. *BMC Bioinformatics*, 8(1):186, 2007.

[5] Thomas E Royce, Joel S Rozowsky, Paul Bertone, Manoj Samanta, Viktor Stolc, Sherman Weissman, Michael Snyder, and Mark Gerstein. Issues in the analysis of oligonucleotide tiling microarrays for transcript mapping. *Trends in Genetics: TIG*, 21(8):466–475, August 2005.

[6] L Stein, P Sternberg, R Durbin, J Thierry-Mieg, and J Spieth. WormBase: network access to the genome and biology of *Caenorhabditis elegans*. *Nucleic Acids Research*, 29(1):82–6, 2001.

[7] Arian R. van Erkel and Peter M. Th. Pattynama. Receiver operating characteristic (ROC) analysis: Basic principles and applications in radiology. *European Journal of Radiology*, 27(2):88–94, May 1998.
